# The effects of ketogenic dieting on skeletal muscle and fat mass

**DOI:** 10.1186/1550-2783-11-S1-P40

**Published:** 2014-12-01

**Authors:** Jacob T Rauch, Jeremy E Silva, Ryan P Lowery, Sean A McCleary, Kevin A Shields, Jacob A Ormes, Matthew H Sharp, Steven I Weiner, John I Georges,, Jeff S Volek, Dominic P D’agostino, Jacob M Wilson

**Affiliations:** 1 The University of Tampa, Tampa, Florida, USA; 2 The Ohio State University, Columbus, Ohio, USA; 3 University of South Florida, Tampa, Florida, USA

## Background

Very low carbohydrate (<5 %), high fat (>70 %) ketogenic diets (VLCKD) diets have previously been shown to have favorable changes in body composition (increased lean mass and decreased fat mass) in obese or overweight individuals. However, the impact of this form of dieting relative to a traditional high carbohydrate diet has not yet been investigated in resistance trained athletes. PURPOSE: Therefore the purpose of this study was to investigate the impact of VLCKD verses a traditional western diet on changes in muscle and fat mass.

## Methods

Twenty-six college aged resistance trained men volunteered to participate in this study and were divided into VLCKD (5 % CHO, 75 % Fat, 20 % Pro) or a traditional western diet (55 % CHO, 25 % fat, 20 % pro). All subjects participated in a periodized resistance-training program three times per week. Body fat and lean mass were determined via dual xray absorptiometry (DXA), while muscle mass was determined via ultrasonography analysis of the quadriceps. All measures were taken at week 0 and 11. Consent to publish the results was obtained from all participants.

## Results

Lean body mass increased to a greater extent in the VLCKD (4.3 ± 1.7 kgs ) as compared to the traditional group (2.2 kg ± 1.7). Ultrasound determined muscle mass increased to a greater extent in the VLCKD group (0.4 ± 0.25 cm) as compared to the traditional western group (0.19 ± 0.26 cm). Finally fat mass decreased to a greater extent in the VLCKD group (-2.2 kg ± 1.2 kg) as compared to the (- 1.5 ± 1.6 kg).

## Conclusions

These results indicate that VLCKD may have more favorable changes in LBM, muscle mass, and body fatness as compared to a traditional western diet in resistance trained males.

